# New programs for translating research to patient care: Lessons learned at the NIH Center for Accelerated Innovations at Cleveland Clinic

**DOI:** 10.1017/cts.2021.849

**Published:** 2021-09-13

**Authors:** Ofer Reizes, Mark Low, Vara Prasad Josyula, James Ellis, D. Geoffrey Vince

**Affiliations:** 1 Department of Cardiovascular and Metabolic Sciences, Lerner Research Institute, Cleveland Clinic, Cleveland, OH, USA; 2 NIH Center for Accelerated Innovations at Cleveland Clinic, Lerner Research Institute, Cleveland Clinic, Cleveland, OH, USA; 3 Proof of Concept Office, Lerner Research Institute, Cleveland Clinic, Cleveland, OH, USA; 4 Department of Biomedical Engineering, Lerner Research Institute, Cleveland Foundation, Cleveland, OH, USA

**Keywords:** Research translation, technology commercialization, investigator education, biomedical innovations

## Abstract

The NIH Center for Accelerated Innovations at Cleveland Clinic (NCAI-CC) was funded by the National Heart Lung and Blood Institute (NHLBI) to support academic investigators in technology development and commercialization. NCAI-CC was one of three multi-institutional Centers established in the fall of 2013. The goal of each Center was to catalyze the growth of an ecosystem of commercialization within their affiliated institutions and regions by managing a program of funding and guiding translational project development and by delivering commercialization education programs to participating investigators. NCAI-CC created and managed such a funding program, ultimately supporting 75 different projects across seven separate academic institutions and developed tailored educational content following the National Science Foundation I-Corps™ curriculum and delivered the program to 79 teams from 12 institutions. We determined early on that in establishment and implementation of projects, it is important to support the teams and principal investigators throughout the program. The support includes a change in principal investigator mindset from specific aims orientation to goals and deliverables on projects. Our skills development efforts emphasized commercialization and a deep understanding of customer needs for new technology adoption. Here, we review our experiences, outcomes, and insights, including the challenges identified in program implementation.

## Organization and Focus of NCAI-CC

**Fig. 1.**
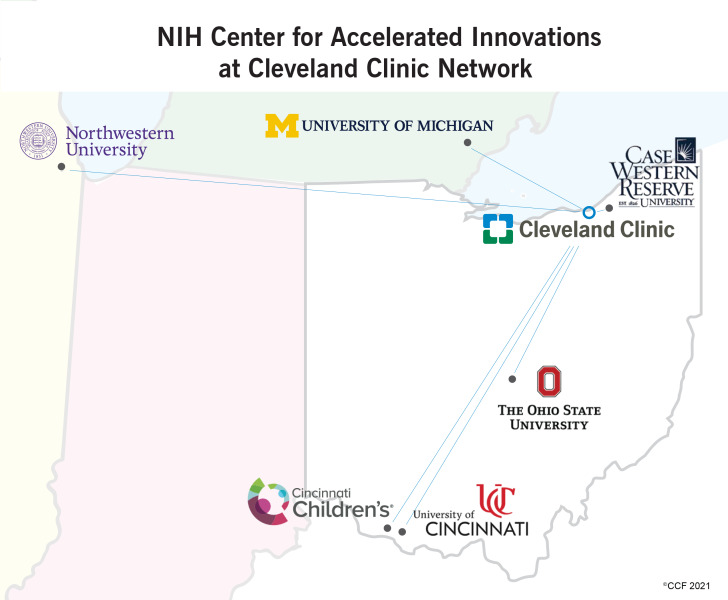
Map of NIH Center for Accelerated Innovations at Cleveland Clinic (NCAI-CC) partners in Midwest. Inaugural consortium was limited the five Ohio-based institutions. Once the systems were in place, the program was scaled to include University of Michigan and Northwestern.

Cleveland Clinic, along with partnering institutions Case Western Reserve University, The Ohio State University, Cincinnati Children’s Hospital Medical Center, the University of Cincinnati, the University of Michigan, and Northwestern University, comprise the NIH Center for Accelerated Innovations at Cleveland Clinic (NCAI-CC, the Center; Fig. 1), under the sponsorship of the NIH National Heart, Lung, and Blood Institute (NHLBI). Program sponsorship by NHLBI focused attention on selecting projects from these three clinical application areas plus sleep disorders which fall under NHLBI’s domain. Projects of interest spanned different technology types including, devices, pharmaceutical and biologic therapeutics, diagnostic assays and systems, and research tools. Center programming was directed equally to all seven institutions with Cleveland Clinic providing coordination of activities. NCAI-CC applied a centralized operating approach, whereby the Center’s program and administrative activities were managed by dedicated personnel employed by Cleveland Clinic. At each partner institution, senior research and/or clinical leaders served as site directors/liaisons, and technology transfer office (TTO) personnel work closely along with Cleveland Clinic managers to promote program activities locally.

A fundamental element of the organizational strategy and operating approach of NCAI-CC was to leverage the State-of-Ohio funded Global Cardiovascular Innovation Center (GCIC) program which was established and operated by Cleveland Clinic from 2007 through 2019. The GCIC organization, comprised of a team of industry-experienced product developers and program managers, had a well-established track record of soliciting, selecting, funding, and guiding development of cardiovascular projects all throughout the state of Ohio. GCIC’s focus, however, was on funding technologies that were being developed by early-stage companies, not projects that were being pursued within academic research or clinical institutions, to achieve economic development objectives of investment and jobs growth. The program was by all accounts successful, but it did not address the significant need for providing similar commercialization-oriented funding, industry-experienced guidance, and education to research institution-based investigators. However, the NCAI opportunity was directed exactly at that gap and with a focus on closely aligned cardiovascular, lung, and blood clinical areas. As such it provided an attractive fit and opportunity for expansion.

GCIC and NCAI-CC operated together as two very synergistic and complementary programs with highly leveraged resources. Through NCAI, we dedicated product development and education programs directed to investigators and early-stage translational projects in the consortium institutions. NCAI augmented the project director staffing with individuals that have more in-depth experience in diagnostics and therapeutic drug development to address more specifically those types of development projects.

## Cultivating, Selecting, and Managing Projects for Development

Our program established a regular semi-annual technology solicitation, selection, and funding process targeting projects that aligned with the NHLBI domain that included heart, lung, blood, and sleep disorders, but which excluded certain disease states that fell under other NIH institutes such as cancer, digestive disease, neurologic disease, and diabetes.

**Fig. 2.**
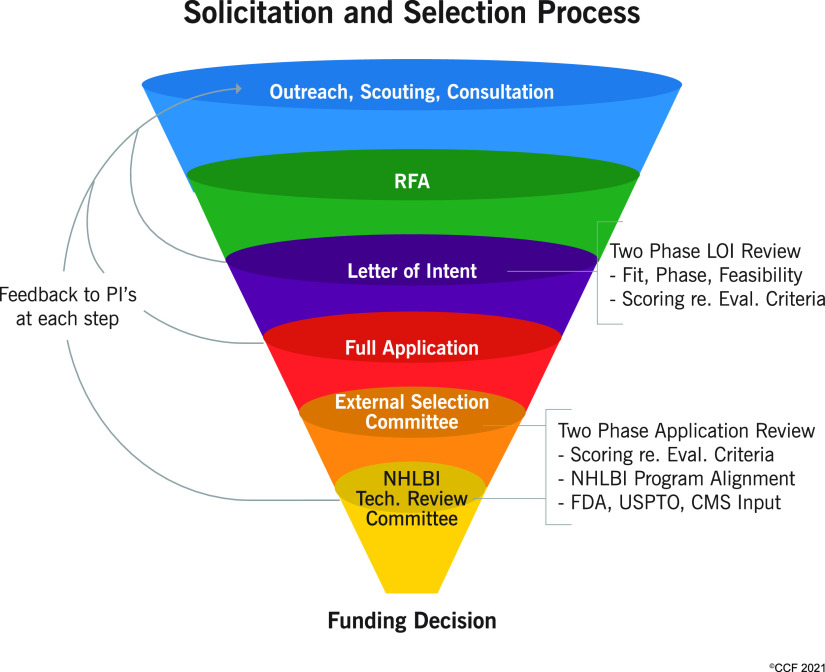
Project selection process. NCAI-CC utilized multi-layered strategy to identify, develop, and fund projects. Overall success rate for projects from 400 letters of intent to the 75 funded proposals was nearly 19%. Abbreviations: CMS, Centers for Medicare and Medicaid Services; FDA, Food and Drug Administration; LOI, letter of intent; NCAI-CC, NIH Center for Accelerated Innovations at Cleveland Clinic; NHLBI, National Heart Lung and Blood Institute; PI, principal investigator; RFA, Request for Application; USPTO, United States Patent and Trademark Office.

Project selection and funding were managed via a Request for Application (RFA) and review process, similar to grant funding processes with which investigators are well familiar for their research (Fig. 2). The prescribed content of the proposals, however, was specific to commercialization-relevant criteria, and very different from research grants. The project description specified sections on scientific and clinical background, unmet need, proposed product/solution, market opportunity, competitive landscape, intellectual property, product value proposition, clinical and regulatory path, payment and reimbursement path, project plan with definitive product development milestones, and commercialization strategy. This was not the usual research proposal aims and research plan content. Since these components are somewhat unique to this translational grant application, writing the proposal itself was a learning process for the Principal Investigators and several investigators mentioned how they can see a bigger picture in terms of product development. At program implementation, this was a major mindset change for applicants and took a significant communication effort on the part of NCAI program and project managers to educate applicants on required project alignment with the program, stage of commercialization readiness and opportunity, and scope of work that would be appropriate for the project.

Project selection was based on 1) fit of the project within the clinical domain areas of NHLBI, 2) the project phase – being to conduct development-oriented activity to achieve commercially relevant milestones, and 3) the feasibility of being able to accomplish the proposed work within the appropriate timeframe and budget. Reflecting that the institutions comprising NCAI-CC were a mix of clinical and university (academic) research institutions, funded Principal Investigators were a mix of researchers (PhDs, 48%), physicians (MDs, 28%), both (MD-PhDs, 20%), or other (4%); however, applications for funding were not evaluated on Principal Investigator education, academic position, or commercialization experience per se. Participating institutions exercised some influence on project eligibility a) by providing the required project matching funds, and b) by providing technology commercialization office support for establishing intellectual property protection and licensing.

Projects that passed the initial Letter of Intent review were invited for full application submission. Applications were reviewed by an External Selection Committee (ESC) comprised of prominent clinicians, scientists, industry representatives, business development experts, and members of the venture capital community, who evaluated the projects on relevant clinical, technical, and commercialization criteria in a study section style review. The proposals were reviewed by at least three ESC members, discussed and ranked, and the most meritorious projects were advanced for a final evaluation by a Technology Review Committee (TRC) convened by NHLBI. The TRC was comprised of representatives from the Food and Drug Administration (FDA), the U.S. Patent and Trademark Office (USPTO), The Centers for Medicare and Medicaid Services (CMS), a major healthcare system, and the NHLBI resident regulatory expert, entrepreneur-in-residence, and investor-in-residence. This review provided unique perspective and valuable feedback to the applicants by key decision-makers in regulatory, commercialization, payment, and healthcare delivery aspects of commercialization. Investigators received instructive feedback on evaluation of their projects at each phase of selection.

The NCAI-CC application process was conducted on semi-annual basis, typically took 24 weeks from RFA to funding announcements (Table 1), and consisted of 10 steps from RFA to project funding/completion.

**Table 1.** Steps to funding from RFA to funded project and implementation

Step	Activity	By	Time
1	RFA distribution (January and July)	NCAI-CC	
2	Letters of intent submission	Applicants	5 weeks
3	Full proposal applicants selected	NCAI-CC	4 weeks
4	Full applications submission	Applicants	6 weeks
5	External selection committee review and meeting	NCAI-CC	3 weeks
6	Recommended applications submitted to NHLBI Technology Review Committee (TRC)	NCAI-CC	1 week
7	NHLBI TRC feedback to NCAI-CC	NHLBI TRC	4 weeks
8	Funding announcements	NCAI-CC	1 week
9	Project period start	Awardee	3 weeks
10	Project period end	Awardee	52 weeks

Abbreviations: NCAI-CC, NIH Center for Accelerated Innovations at Cleveland Clinic; NHLBI, National Heart Lung and Blood Institute; RFA, Request for Application; TRC, Technology Review Committee.

NCAI project directors from Cleveland Clinic engaged with prospective and funded investigators on a continuous basis throughout the program. Some of the principal investigators, upon request, had an opportunity to discuss their technology with the NHLBI regulatory expert to understand the process on a first-hand basis. This engagement was not limited to the funding round periods when active solicitation of applications was ongoing, but was a consistent engagement with all the partner institutions to scout for and cultivate candidate projects and to work with the investigators to make for more compelling applications. This involved, for example, consulting on doing the necessary research, developing lead compounds, collecting relevant data such as in vitro/in vivo efficacy data, and discussing commercialization strategy. Engagement continued throughout the funded project period – keeping focus on reaching the project milestones and extended beyond to advising on post-project continued development and commercialization activities.

## Education and Entrepreneurial Development

A major objective of the NHLBI NCAI mission was implementation of an entrepreneurship education and skills development program within each of the Centers. From the beginning, NCAI-CC focused on providing direct skills development instruction to the teams from all partner institutions through individual engagement by the Center’s project development directors. The goal of the education program was to provide investigators with commercialization-focused skills that complemented the proposal solicitation. Soon after program implementation, the efforts were enhanced with the introduction of NSF I-Corps™. NHLBI partnered with the National Science Foundation (NSF) to support implementation of I-Corps™ as a component of NCAI skills development initiatives and facilitated a special funding opportunity whereby NCAI Centers could apply for funding to establish I-Corps Hubs within their programs. The I-Corps entrepreneurial education program was designed to identify the customer needs and model the critical steps for business development. The NSF program has seen various iterations since early development and the NCAI-CC program adopted basic features while piloting different strategies to specifically tailor the I-Corps content for the unique needs of biomedical investigators. We rolled out I-Corps@CC to Cleveland Clinic and Case Western Reserve University. The program introduced biomedical-tailored I-Corps lectures on all segments of the business model canvas delivered by industry-experienced entrepreneurs and project managers, plus a significant library of supplemental readings focused on specific biomedical project considerations. The specialized topics are defined here and were also incorporated into NCAI-funded project kick-off meetings:

- Value Creation in Healthcare Product Development and Commercialization
- Intellectual Property (IP) and Building Value through IP
- Target Product Profiles and Workflows
- Understanding Your Regulatory Pathway
- Medical Market Segmentation
- Key Commercialization Activities and Resources
- Partnerships, Relationships, and Channels
- Cost, Reimbursement, and Payers
- MedTech Reimbursement for Entrepreneurs

The goal of the skills development program was implementation of entrepreneurial educational programs to investigators throughout the consortium of NCAI-CC partner institutions. To expand the implementation of I-Corps@CC to the other NCAI-CC partner institutions, we partnered with the State of Ohio NSF-sanctioned I-Corps@Ohio program. The Ohio State University was funded by the Ohio Department of Education to provide the generic I-Corps program to academic investigators at institutions throughout the state. I-Corps@CC was able to complement the I-Corps@Ohio program by providing biomedical-specific content and thereby to extend the reach to all NCAI-CC Ohio partner institutions, as well as to other Ohio institutions, and recently also to the NCATS Clinical Translational Science Collaborative program’s implementation of I-Corps@NCATS. Thus, the Center has been able to augment its direct-to-project team individual coaching engagement by project directors with curriculum-based education on a wider scale.

## Project Results and Commercialization Progress

During the course of the program, NCAI-CC conducted 14 funding rounds, in which nearly 400 letters of intent were submitted; 141 full applications evaluated; and 75 projects awarded funding. Total federal funding awarded to these projects by NCAI was $8.85 Million, to which $5.61 Million of cost share was added by local non-federal sources, yielding an average per project budget of $193,000. Of the 75 projects funded, all but three were successfully completed or are in the process of concluding. The reasons for not completing projects were primarily due to the PI relocating to another institution during the project period.

At time of writing, 36 of the funded projects (48%) progressed to the stage of having been licensed or optioned to newly started or existing companies. Eight of those projects received SBIR or STTR funding to continue development through 15 separate awards. Notably, two projects have later been acquired by major pharmaceutical companies Novo Nordisk and Amgen. Total follow-on funding or financing deals commit $1.3 Billion to the continued development and commercialization of the technologies (dependent on achievement of future milestones). Major factors for success to which these results can largely be attributed include a rigorous, qualification-driven project solicitation and selection process, experienced and commercialization-directed project management, and access to technology validation funding sources to support early-stage development.

## Key Learnings, Challenges, and Best Practices

Over the course of conceiving, designing, operationalizing, and managing a multi-institutional, NIH-funded program to accelerate the translation of research discoveries to development for improving patient care, we learned many lessons, overcame challenges, and believe that we established certain best practices that can be instructive to other programs. Findings along that journey can be grouped into the following categories:

### Development and Commercialization Focus

Perhaps the first challenge and realization were that managing a program focused on product development and commercialization in contrast to academic research and publication focus necessitated a major mindset change in the investigators to whom the program was targeted. Rather than pursuing the concept of research aims, the program was focused on defining and executing commercialization-relevant product development milestones and timelines. Despite the emphasis of these elements in funding RFAs and repeatedly in funding cycle information sessions, early in the program it was not uncommon to receive slightly modified R01 aims pages as letters of intent. It took a significant educational effort over the first several RFA cycles to establish that the program served different objectives, namely directed towards achievement of critical development milestones to advance the technology towards commercialization and to achieve specific measurable project activities that are critical to establishing commercial opportunity. Similarly, to meet stage of readiness criteria, the projects had to have already reached certain stage of development. For example, for therapeutics development projects, it was strongly recommended that a lead compound was already identified with a disease target validated by appropriate screening assays and with initial indication of in vivo efficacy. With progressive funding cycles, these criteria became more familiar within the participating institutions and among the candidate investigators, and applications reflected more mature, development-oriented projects. And a higher percentage got funded.

### Project Management

Project directors employed by the Center played a critical role, both in cultivating potential projects for the program and in working closely with the PIs to support the development work itself. The program project directors were industry-experienced senior product development engineers with either medical device or pharma backgrounds. These experts were deployed to work with all partner institutions in the Center and engaged in all aspects of candidate project identification, qualification, application for funding, and consulting to the project before, during, and after the project period. The result and impact were that candidate projects were very often cultivated with input from the project directors, guided by them during the project period to efficiently achieve targeted development milestones, and often strategically positioned with their input for follow-on funding, investment, and licensing opportunities. Frequently, investigators cited engagement with the project directors as one of the most significant aspects of the program (aside from the funding, of course).

### Project Funding

The program managed federal funding to the investigators via subgrants from the main grant at Cleveland Clinic for one-half of the project direct costs and associated indirect costs, plus the requirement for 1:1 non-federal matching funds for direct costs to be supplied from other, usually institution-managed, sources. The federal funds were distributed on an invoice reimbursement basis and depended on the project making progress towards the stated project milestones and timelines, like a phase-gate process, as reviewed by the project directors. This provided a measure of accountability toward the work conducted, which was different from the way investigators were used to having their projects administered.

Initially, non-federal matching funds were difficult to source. Many of the participating institutions did not have matching funds sources available a) to commit to the project during the application phase as per requirement of the project RFA and b) to be distributed to the project in coordination with the federal funds. Over time, however, the institutions did identify and allocate funding sources for this purpose and aligned the timing of their decision processes to coordinate funding awards from those sources with funding awards from the Center for the approved projects.

The administrative sub-grant process for federal funds from the Center to the project institutions also had a degree of difficulty in coordination throughout the program. This resulted in delays in funds availability for targeted project start dates and consequently for project completion dates. This was an internal issue but reflected a challenge in managing a multi-institutional project funding program. The projects also ran up against NIH annual funding timelines that do not coincide with the project timelines. As such, despite best efforts to manage project timelines and to keep scheduling, these administrative delays resulted in a higher-than-expected incidence of project timeline extension requests.

### Education

NCAI-CC took a primary direct-to-investigators approach to skills and entrepreneurship development. Education provided was dynamic and supported the immediate and specific needs of the investigators and their projects. To complement and expand this, the Center also adopted the NSF I-Corps curriculum and specially tailored the content to support biomedical investigators for broader education. A significant learning from the NCAI-CC program was the critical need for both individualized investigator support in entrepreneurial skills development and specifically focused programs that specialize in building a greater understanding of the value proposition of the technology. Our approach also provided significant engagement with individual investigators as they developed their technology from concept to funded project and follow-on support.

### Program Scalability and Sustainability

A stated objective of the overall NCAI program was that participating centers would develop means to scale and sustain the program beyond the original scope and federal funding of the program. The NCAI program at Cleveland Clinic did successfully demonstrate the ability to expand the number of participating institutions by adding the University of Michigan and Northwestern University to the consortium in year 4 of the program and to extend the centrally provided project management support by the project directors to new institutions. The model of engagement with the new institutions was based on deploying the commercialization-oriented, RFA-driven funding program qualification criteria, along with in-person engagement with the Technology Commercialization offices and with candidate investigators, like with the original institutions. Greater distances from Cleveland presented some logistical adjustments and incurred additional expense but was demonstrated to be workable.

The program has also demonstrated scalability in the education of researchers for technology commercialization objectives by incorporating the specially tailored I-Corps™ biomedical/bioscience curriculum developed under the NSF grant to Cleveland Clinic into the broader I-Corps@Ohio program that reaches well beyond the NCAI participating institutions.

Sustainability of the program can be viewed from at least five perspectives.

- A model for designing and managing academic institution-based technology development and commercialization programs. For example, the State of Ohio has developed and manages a Technology Validation and Start-up Fund that is directed towards similar stage academic research-based development and commercialization, which has very similar qualifying criteria to the NCAI program, and is broader than just biomedical/bioscience applications. It served as the source of matching funds for many of the NCAI funded projects. Coulter Foundation and Fast Forward Innovation programs also operate at participating institutions.
- An approach for incorporating commercialization-relevant expertise into project management at these programs. Industry-experienced project directors/managers and regulatory advisors were already in place or have been added to the technology commercialization organizations at each of the NCAI-CC participating institutions.
- A model for the commercialization education of investigators at academic institutions. As described, the I-Corps curriculum has been specially tailored for biomedical/bioscience applications and is deployed on an ongoing basis to investigators at a dozen Ohio institutions, well beyond the original reach of the program.
- A catalyst for establishing new funds to support early-stage product development at academic institutions. The program originally proposed an objective of re-investing proceeds from licensing or commercialization successes from program-funded projects back into the institutions for deployment to new projects. Such re-investment may well be managed by the individual institutions as their projects progress and yield licensing returns, depending on their policies and practices. However, there are no current means for such proceeds to feed back to the Center for its sustainability per se, and the timelines for such returns on licensing of such early-stage projects are beyond the horizon for sustaining the original program. Nevertheless, each of the participating institutions reports that they have raised new dedicated funding sources to continue and to grow these types of research translation programs.
- The value of including input from the FDA, NHLBI, CMS, USPTO, and other healthcare systems, early in the technology selection process. This feedback not only helped determine which projects were ultimately funded but also helped shape the technology development program to rapidly advance the technologies to a commercial state.

## Conclusion: Impact and Influence

NCAI-CC was a consortium of seven prominent academic research institutions and medical centers spanning the state of Ohio and extending to Michigan and Illinois. Although each operated independently, collectively they had great influence on developing and expanding the research translation and commercialization ecosystem in their regions. Each institution has incorporated elements of the NCAI experience and practices into their ongoing programs of technology commercialization. New sources of technology development funding have been established at institution, regional, state, and federal levels to provide progressive sources and continuity of support for early-stage product development. Entrepreneurship and commercialization education programs have been established and expanded to provide investigators with ongoing resources and direction to guide their product development. Inter-institutional collaboration in these activities has grown, reflective in part of the consortium participation and engagement. All institutions acknowledge that they are stronger and that the ecosystem is more robust for having their participation in the NCAI program.

